# Growth and Biomass Distribution Responses of *Populus tomentosa* to Long-Term Water–Nitrogen Coupling in the North China Plain

**DOI:** 10.3390/plants14121833

**Published:** 2025-06-14

**Authors:** Yafei Wang, Juntao Liu, Yuelin He, Wei Zhu, Liming Jia, Benye Xi

**Affiliations:** 1 Key Laboratory of Silviculture and Conservation of the Ministry of Education, College of Forestry, Beijing Forestry University, Beijing 100083, China; mrw1996@bjfu.edu.cn (Y.W.); ljt1120@bjfu.edu.cn (J.L.); 2 Guangdong Provincial Key Laboratory of Silviculture, Protection and Utilization/Guangdong Academy of Forestry, Guangzhou 510520, China; 3 National Key Laboratory for Development and Utilization of Forest Food Resources, Zhejiang A&F University, Hangzhou 311300, China; hylhelen@163.com; 4 College of Biological and Environmental Engineering, Jingdezhen University, Jingdezhen 333400, China; 18811375082@163.com

**Keywords:** short-rotation plantations, tree growth, biomass allocation, irrigation water use efficiency, nitrogen fertilizer partial productivity

## Abstract

From 2016 to 2021, a field experiment was conducted in the North China Plain to study the long-term effects of drip irrigation and nitrogen coupling on the growth, biomass allocation, and irrigation water and fertilizer use efficiency of short-rotation triploid *Populus tomentosa* plantations. The experiment adopted a completely randomized block design, with one control (CK) and six water–nitrogen coupling treatments (IF, two irrigation levels × three nitrogen application levels). Data analysis was conducted using ANOVA, regression models, Spearman’s correlation analysis, and path analysis. The results showed that the effects of water and nitrogen treatments on the annual increment of diameter at breast height (ΔDBH), annual increment of tree height (Δ*H*), basal area of the stand (*BA*_S_), stand volume (*V*_S_), and annual forest productivity (AFP) in short-rotation forestry exhibited a significant stand age effect. The coupling of water and nitrogen significantly promoted the DBH growth of 2-year-old trees (*p* < 0.05), but after 3 years of age, the promoting effect of water and nitrogen coupling gradually diminished. In the 6th year, the above-ground biomass of *Populus tomentosa* was 5.16 to 6.62 times the under-ground biomass under different treatments. Compared to the I45 treatment (irrigation at soil water potential of −45 kPa), the irrigation water use efficiency of the I20 treatment (−20 kPa) decreased by 88.79%. PFP showed a downward trend with the increase in fertilization amount, dropping by 130.95% and 132.86% under the I20 and I45 irrigation levels. Path analysis indicated that irrigation had a significant effect on the *BA*_S_, *V*_S_, AFP, and TGB of 6-year-old *Populus tomentosa* (*p* < 0.05), with the universality of irrigation being higher than that of fertilization. It is recommended to implement phased water and fertilizer management for *Populus tomentosa* plantations in the North China Plain. During 1–3 years of tree age, adequate irrigation should be ensured and nitrogen fertilizer application increased. Between the ages of 4 and 6, irrigation and fertilization should be ceased to reduce resource wastage. This work provides scientific guidance for water and fertilizer management in short-rotation plantations.

## 1. Introduction

The total amount of natural forest land worldwide declined more sharply, by almost 301 million hectares, over the thirty years between 1990 and 2020. Nonetheless, there was an increase of 123 million hectares in the amount of planted forests. Though the demand for lumber is still growing globally, the amount of timber produced by natural forests worldwide has already reached its peak [1]. Therefore, to resolve the current supply–demand imbalance in the international timber market, the foremost priority is to improve the yield and quality of plantation forests through sustainable management strategies that are effective, scientific, and rational, which requires comprehensive research on the long-term effects of various intensive cultivation methods on the ecological functions of plantations [2,3,4].

In the present day, the ingenious integration of irrigation and fertilization has become an established developmental path for achieving water–fertilizer integration, as well as a critical pathway for resolving the issues of irrational water and fertilizer application [5,6]. Water and fertilizer coupling has the distinct advantage of leveraging water to improve fertilizer efficacy while also utilizing fertilizer to control water usage. As a result, among the numerous significant measures for intensive plantation management, promoting tree growth through water–fertilizer coupling frequently outperforms the results achieved by implementing irrigation or fertilization alone [7,8,9,10]. The overall effect of water and fertilizer synergy is proportional to their ratio and varies with changes in each component. Under situations of adequate soil moisture, the interaction between water and fertilizer can have a superposition effect, promoting plant growth [11,12,13]. When soil moisture is in a restricted state, excessive application of nitrogen fertilizer not only fails to promote plant growth but also produces antagonistic effects [14,15]. In plantation cultivation, a thorough understanding of tree growth mechanisms is critical for accomplishing long-term forest resource management. The dynamic process of tree growth refers to the patterns of changes in radial growth, height, and overall volume of trees as they mature. Investigating these dynamic processes allows us to understand the underlying laws of stand growth. Understanding tree development patterns under long-term water and nitrogen coupling is very useful for developing dynamic irrigation and fertilization methods during the rotation period [16,17]. According to our understanding, there are still few ongoing studies and publications on the long-term impacts of water and nitrogen [8,9,18].

During the growth and development process, trees prioritize the allocation of biomass to organs capable of acquiring limited resources, adjusting to the environment through constant optimization and modification [19]. A logical biomass allocation scheme is critical for forest tree growth because it can significantly increase growth rate and survival capacity [20]. The logical allocation of above-ground biomass (AGB) can improve light energy usage efficiency, boost photosynthesis, and maximize radial and height development [21,22]. Meanwhile, below-ground biomass (BGB) is essential for plants to compete for limited soil nutrient resources [23,24]. This makes it obvious that the distribution of biomass is intimately linked to the forest tree growth process and is a valuable method and instrument for identifying forest tree growth strategies. Nonetheless, there are still several obstacles in the field of biomass research today. The majority of research has concentrated mostly on the aboveground portions because biomass sampling is very challenging, particularly for belowground biomass. The allocation patterns of various root organs are frequently overlooked, even in studies that focus on the subsurface [22,24,25]. Because of this, we are still unable to evaluate the growth patterns and environmental adaptation of many tree species, especially broad-leaved wood species that have the capacity to develop. Meanwhile, biomass is a fundamental component of forest growth and a key indicator of plantation productivity. The distribution of biomass among tree organs reflects their adaptive methods in growth space. This allocation pattern represents the trade-offs that trees make in the acquisition, accumulation, usage, and distribution of resources [23,26,27]. On the one hand, it is controlled by the species and genotype of trees, and the distribution of biomass may change significantly among tree species [24,25,27,28]. However, according to the functional balance theory and the optimal allocation theory, external environmental factors (like forest age, light, water, and nutrients) also have a significant impact on the patterns of biomass allocation [9,19,29,30,31,32,33]. Therefore, it is crucial to advance the theoretical and practical innovation of plantation cultivation techniques by carrying out a comprehensive study on the biomass allocation patterns of forest trees and resolving the limitations of previous studies.

Poplar (*Populus* spp.) is grown extensively around the world; professional poplar plantations span about 9.4 million hectares, mostly in China, Canada, and Europe. In addition to its many beneficial qualities, such as its propensity for hybridization and ease of propagation [34], it is essential for the world’s timber supply, shelterbelt construction, energy provision, carbon sequestration, degraded land restoration, and soil and water conservation [3,34]. In China, poplar plantations account for 6.54% of the timber forest area, yet they contribute to nearly 30% of the country’s timber production [3]. Poplars in China are primarily planted in areas with poor site conditions, with the white poplar (*Populus tomentosa*) often cultivated on sandy soils in the Yellow River floodplain region. Water availability is a crucial limiting factor for their growth because they are a species that uses a lot of water [34]. Furthermore, prior research has demonstrated that poplar trees have a greater preference for nitrogen than other fertility elements, making nitrogen the main factor restricting the growth of both trees and crops in northern China [8,9,35]. It appears that extensive water and nitrogen management strategies are crucial for increasing the productivity and quality of poplar plantations in northern China, as well as mitigating the negative impacts of water scarcity on tree development and fitness.

A five-year irrigation and fertilization study was conducted in *Populus tomentosa* plantations in the North China Plain to increase poplar productivity and evaluate the effects of water and nitrogen coupling on plant production. The objectives of this study were to (1) investigate the long-term response of *Populus tomentosa* growth processes to water–nitrogen coupling measures during the rotation period; (2) investigate the allocation patterns of *Populus tomentosa* biomass at the end of the rotation under long-term water–nitrogen coupling; (3) determine whether water–nitrogen coupling could change the water use efficiency and nitrogen fertilizer partial productivity of *Populus tomentosa* over the entire rotation period. This study will serve as a reference for the high-yield, scientific, and sustainable management of worldwide short-rotation plantations.

## 2. Results

### 2.1. Long-Term Effects of Different Water–Nitrogen Coupling Treatments on Tree Growth

**Figure 1:**
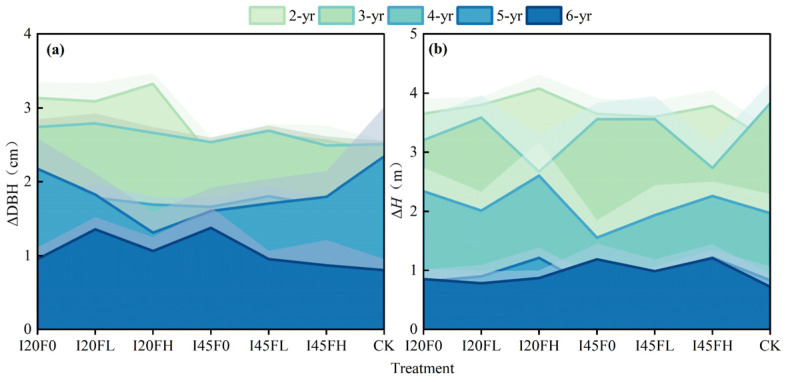
Effects of different treatments on the annual increment of diameter at breast height (**a**) and the annual increment of tree height (**b**) in 2–6-year-old *Populus tomentosa* stands. Note: The gray shaded area represents the standard error.

For individual *Populus tomentosa* trees, compared to the control treatment, the water–nitrogen coupling treatment significantly increased ΔDBH at two years of age, with the I20FH treatment showing the greatest increase, 37.30% higher than the CK treatment (*p* < 0.05). The difference in ΔDBH under different treatments gradually decreased as the trees grew to 3 to 6 years old (Table 1). Throughout the investigation, the water–nitrogen coupling treatment had no significant influence on tree height (Table 1). As stand age increased, ΔDBH and Δ*H* decreased annually (Figure 1). The difference in irrigation levels was reflected in the DBH increment at 2 years old; the fertilization effect was manifested in the height increment at 5 years old. No significant interaction effect between irrigation and fertilization was observed in the annual DBH and height increments (Figure 1 and Table 1).

**Figure 2:**
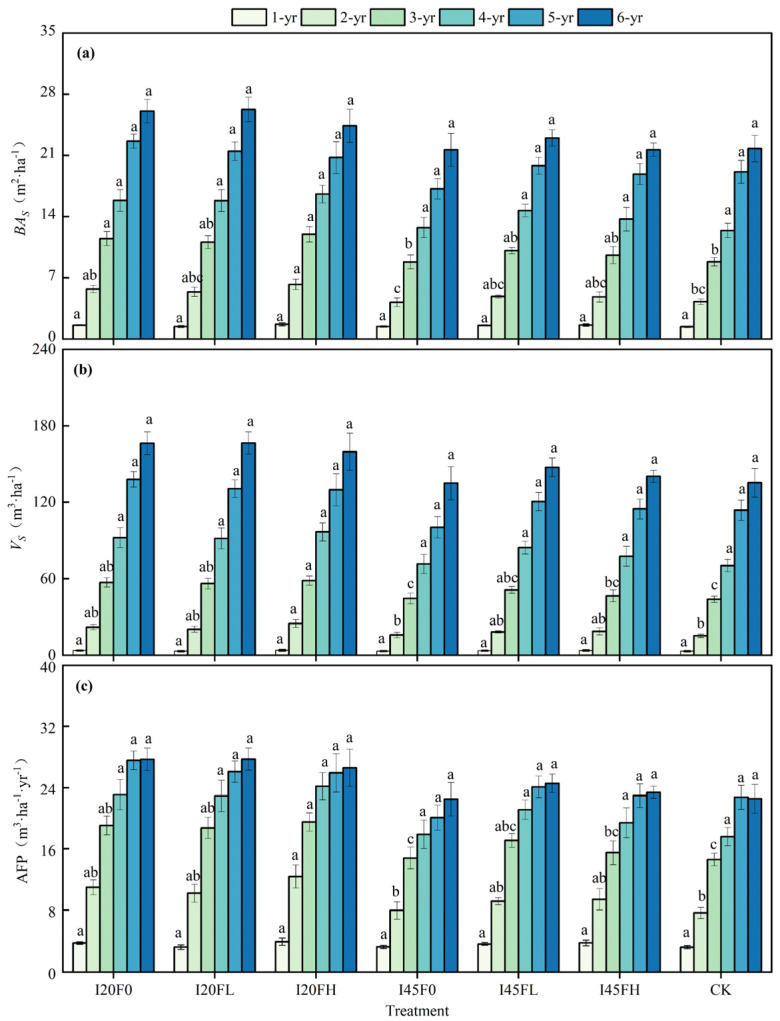
Effects of different treatments on stand basal area (**a**), stand volume (**b**), and average forest productivity (**c**) for 2–6-year-old *Populus tomentosa* stands. Note: Each value represents the mean of five replicates, with error bars indicating the standard error of the mean. A one-way ANOVA was used to test the effects of different treatments on stand growth indices within the same stand age, and multiple comparisons were conducted using a Duncan’s test. Different lowercase letters indicate significant differences among treatments within the same year.

The *BA*_S_ of the 2–3-year-old water–nitrogen coupling treatments differed significantly from the CK treatment (Table 1). Except for the I45F0 treatment, the remaining five water–nitrogen treatments enhanced the stand’s basal area. Compared to the CK treatment, the I20FH treatment significantly increased *BA*_S_ by 46.57% and 35.80% at 2 and 3 years of age, respectively (*p* < 0.05) (Figure 2). At 4–6 years old, no significant variations in *BA*_S_ were found between treatments (Figure 2 and Table 1). The therapies had similar impacts on *Vs* and AFP as they did on *BA*_S_. However, these significant differences did not show until 3 years old. Among the six water–fertilizer treatments, the *BA*_S_, *V*_S_, and AFP of the 2–6-year-old forest stand responded strongly to irrigation (*p* < 0.05) but were unaffected by fertilization or interaction effects.

**Figure 3:**
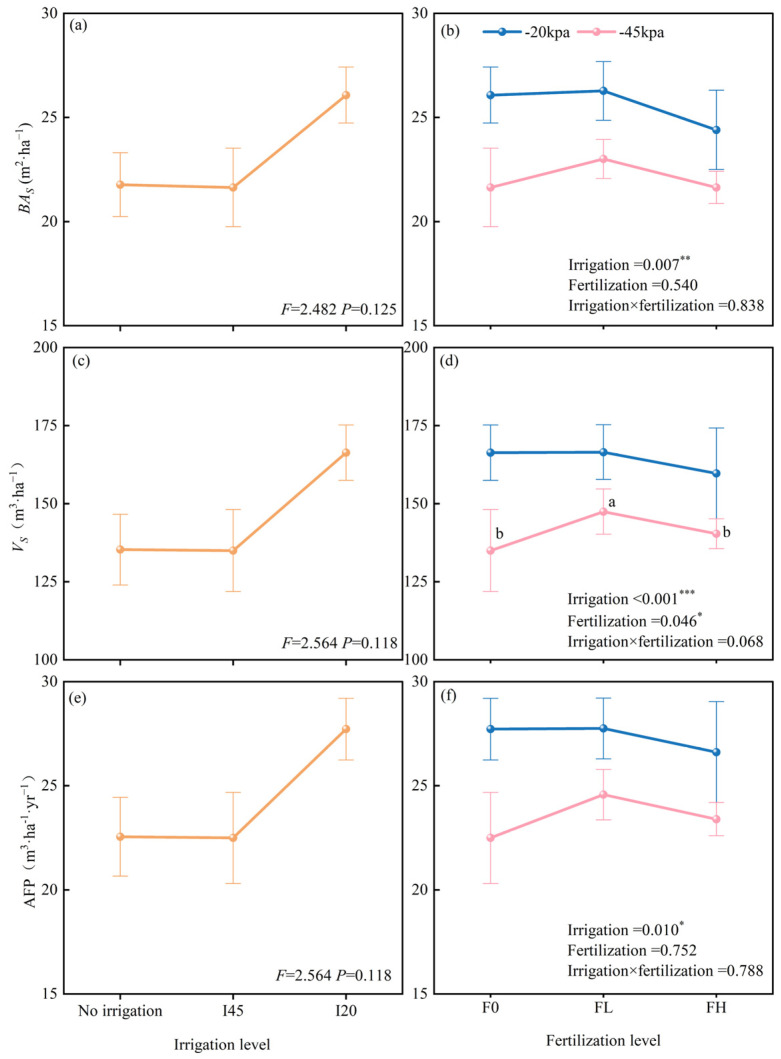
Effects of different irrigation gradients and fertilization levels on stand basal area (**a**,**b**), stand volume (**c**,**d**), and mean site productivity (**e**,**f**). Note: Each value represents the mean of five replicates, with error bars indicating the standard error of the mean. A two-way ANOVA was used to analyze the effects of irrigation level, fertilization level, and their interaction on various stand growth indicators. A one-way ANOVA was employed to test the effects of different irrigation levels and different fertilization levels under the same irrigation level on stand growth indicators, with multiple comparisons conducted using a Duncan’s test. * indicates *p* < 0.05, ** indicates *p* < 0.01, *** indicates *p* < 0.001. Different lowercase letters indicate differences under various irrigation/fertilization levels.

In the sixth year, there were no significant variations in *BA*_S_, *V*_S_, or AFP between the unfertilized treatments under different irrigation gradients (I20F0, I45F0, and CK). The same was true for the different fertilized treatments under high irrigation (I20F0, I20FL, and I20FH). However, for the low irrigation treatments (I45F0, I45FL, and I45FH), we found that *Vs* increased initially and then dropped as fertilization amount increased (Figure 3).

### 2.2. Long-Term Effects of Different Water–Nitrogen Coupling Treatments on Biomass Distribution Pattern

**Figure 4:**
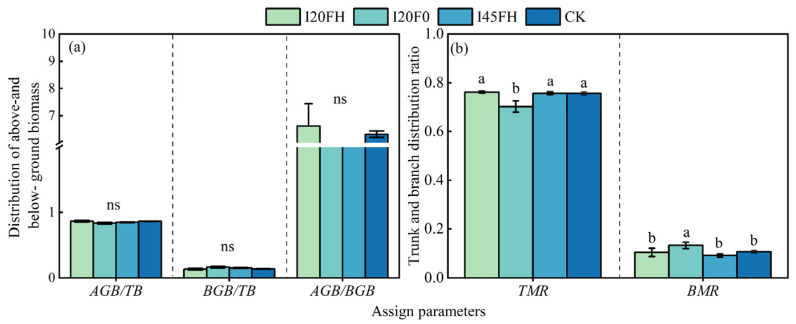
Effects of different treatments on the allocation ratio of above-ground to below-ground biomass (**a**) and the allocation ratio of biomass in stem and branch organs (**b**). Note: Each value represents the mean of three replicates, with error bars indicating the standard error of the mean. A one-way analysis of variance (ANOVA) was used to assess the effects of treatments on individual tree biomass allocation, followed by multiple comparisons using a Duncan’s test. Different lowercase letters indicate significant differences between treatments.

**Figure 5:**
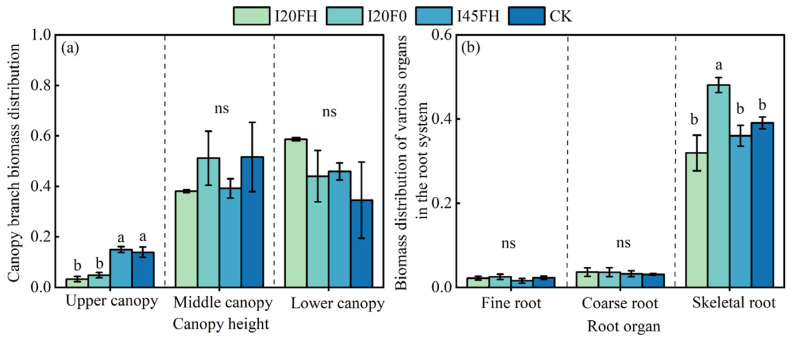
Effects of different treatments on the biomass allocation ratio of canopy branches (**a**) and the biomass allocation ratio of various root organs (**b**). Note: Each value represents the mean of three replicates, and the error bars indicate the standard error of the mean. A one-way ANOVA was used to test the effects of different treatments on individual tree biomass allocation, and multiple comparisons were conducted using a Duncan’s test. Different lowercase letters indicate significant differences between treatments.

In the sixth year, there was no significant difference in aboveground and belowground biomass allocation among treatments (*p* > 0.05). The above-ground biomass allocation exceeded the below-ground biomass allocation by 4.16 to 5.62 times (Figure 4a). The biomass allocation between trunks and branches varied among treatments, with full irrigation without fertilization reducing trunk biomass allocation (−5.37%) while boosting branch biomass allocation (+2.58%) (Figure 4b). Upper canopy branches (3.30~14.96%) had significantly reduced allocation compared to middle and lower canopy branches (34.51%~58.64%), with significant variations between treatments (*p* < 0.05) (Figure 5a). The low water irrigation (I45) and no irrigation (CK) treatments had considerably larger proportions of top canopy branches compared to the high water irrigation treatment (I20) (*p* < 0.05) (Figure 5a). Different root organs reacted differently to the treatments. Fine and coarse roots responded weakly to diverse treatments, with no significant differences between them (*p* > 0.05) (Figure 5b). The I20F0 treatment had a considerably larger allocation ratio for skeletal root biomass compared to the I20FH, I45FH, and CK treatments (*p* < 0.05) (Figure 5b).

**Figure 6:**
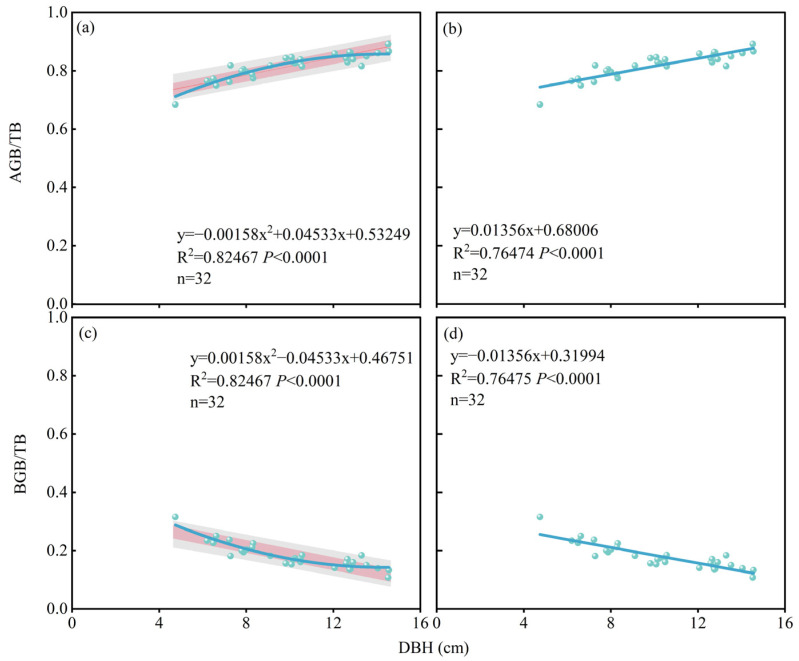
Linear and quadratic regression models of the proportion of aboveground biomass (**a**,**b**) and belowground biomass (**c**,**d**) of forest trees in relation to diameter at breast height (n = 32). Note: The pink shading represents the 95% prediction band, and the gray shading represents the 95% confidence band.

For the above-ground portion, the corresponding quadratic and linear regression models were y = 0.00158x^2^ + 0.04533x + 0.53249 (*R*^2^ = 0.82, *p* < 0.001) and y = 0.01356x + 0.68006 (*R*^2^ = 0.76, *p* < 0.001). For the below-ground portion, the corresponding quadratic and linear regression models were y = 0.00158x^2^ − 0.04533x + 0.46751 (*R*^2^ = 0.82, *p* < 0.001) and y = −0.01356x + 0.31994 (*R*^2^ = 0.76, *p* < 0.001). When the models’ *R*^2^ values are compared, the quadratic regression model surpasses the linear regression model and more closely reflects our observed data. Using the quadratic model, we discovered that when the *Populus tomentosa* diameter is small (younger in age), the allocation proportion to the aboveground part of the *Populus tomentosa* increases rapidly, while the below-ground allocation proportion declines. However, when the *Populus tomentosa*’s diameter rises, this proportion progressively stabilizes, and as the *Populus tomentosa* becomes larger, there is a pattern of diminishing aboveground allocation and increasing below-ground biomass allocation (Figure 6a,c).

### 2.3. Long-Term Effects of Different Water–Nitrogen Coupling Treatments on WUE and PFP

**Figure 7:**
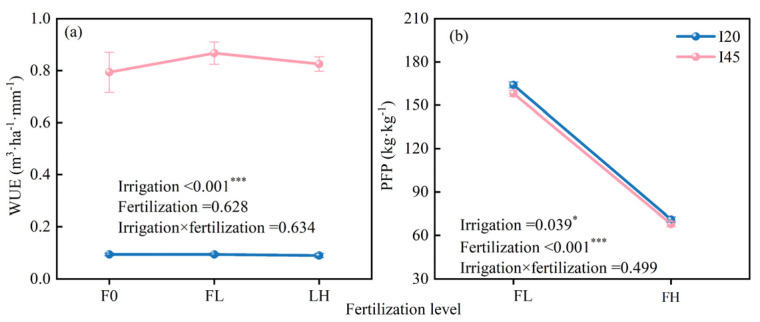
Effects of different irrigation gradients and fertilization levels on stand water use efficiency (WUE) (**a**) and partial factor productivity of nitrogen fertilizer (PFP) (**b**). Note: Each value represents the mean of five replicates, with error bars indicating the standard error of the mean. A two-way ANOVA was employed to analyze the effects of irrigation level, fertilization level, and their interaction on WUE and PFP. * indicates *p* < 0.05, *** indicates *p* < 0.001.

In the sixth year, irrigation level had a significant effect on WUE, which was significantly lower in the I20 treatment than in the I45 treatment, with an average reduction of 88.79% under the three fertilization levels (*p* < 0.001) (Figure 7a). Fertilization with irrigation did not significantly promote WUE (*p* > 0.05) (Figure 7a), but there was a potential for WUE to initially increase and then decrease with increasing fertilization, which was more pronounced in the I45 treatment compared to the I20 treatment. Irrigation and fertilization levels had a substantial impact on PFP (*p* < 0.05) (Figure 7b). PFP showed a decreasing trend with the increase in fertilization level, with reductions of 130.95% and 132.86% for the I20 and I45 treatments, respectively. (Figure 7b).

### 2.4. Correlation and Path Analysis

**Figure 8:**
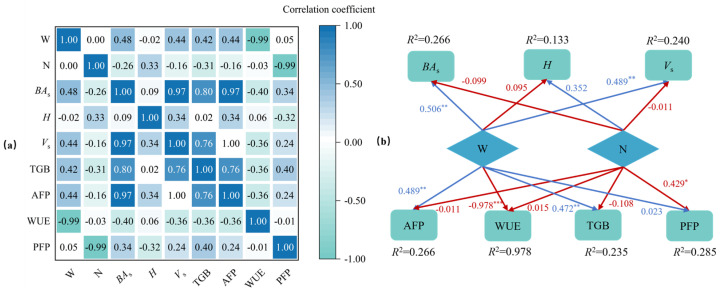
Correlation analysis (**a**) and path analysis (**b**) of irrigation (W) and fertilization (N) gradients with tree height (*H*), stand basal area (*BA*_S_), stand volume (*V*_S_) and productivity (AFP), forest biomass (TGB), water use efficiency (WUE), and partial factor productivity of nitrogen fertilizer (PFP). Note: Blue arrows represent positive effects; red arrows represent negative effects. *** indicates *p* < 0.001; ** indicates *p* < 0.01; * indicates *p* < 0.05.

Correlation analysis showed that irrigation levels were considerably negatively connected with WUE (*p* < 0.001) (Figure 8a and Table S1) and strongly correlated with *V*_S_, TGB, and AFP (*p* < 0.01) (Figure 8a and Table S1), with correlation values of 0.44, 0.42, and 0.44, respectively. Irrigation had a negative effect on WUE, with a direct path coefficient of -0.978 (*p* < 0.01) (Figure 8b), but had substantial beneficial effects on *BA*_S_, *V*_S_, AFP, and TGB, with direct path coefficients of 0.506, 0.489, 0.489, and 0.472, respectively. The fertilization level had a strong connection with PFP (*p* < 0.01) (Table S1), with a correlation coefficient of 0.99 (Figure 8a). The fertilization level had a negative impact on *BA*_S_, *V*_S_, AFP, TGB, WUE and PFP, and only a positive impact on *H*. Among the direct effects on various indicators, only PFP reached a significant level, with a direct path coefficient of 0.429 (*p* < 0.01) (Figure 8b). It is clear that irrigation exerts a more consistently beneficial influence on *Populus tomentosa* growth than fertilization.

## 3. Discussion

### 3.1. Long-Term Effects of Water–Nitrogen Coupling on Tree Growth

The productivity of forest land can be raised and tree growth accelerated by fertilizer and irrigation. However, most studies were often confined to specific stand ages, ignoring the different responses to fertilization and irrigation across various developmental stages within the forest rotation cycle [7,8,9,36,37,38]. The growth of short-rotation *Populus tomentosa* forest in this study showed an age-dependent response to various nitrogen and water treatments (Figure 1 and Figure 2). Regarding *Populus tomentosa*, we discovered that the water–nitrogen coupling considerably increased the DBH development in trees that were two years old, but the response to the water–nitrogen coupling progressively ceased to be significant after three years of age (Figure 1 and Table 1). The variation in responses of different-aged *Populus tomentosa* to water–nitrogen coupling is primarily influenced by changes in tree living space. Irrigation and nitrogen application can hasten the development of the *Populus tomentosa*’s underground portions during the early phases of growth. But as the trees grow larger, the living space of forest trees increasingly limits the expansion of their underground portions, and their root systems gradually start to grow vertically along the soil profile. *Populus tomentosa* is a tree species with fine roots that can reach close to the groundwater level, according to earlier research [39,40]. The North China Plain is dominated by agricultural production, where fertilizer application frequently surpasses crop requirements. This results in elevated levels of NO_3_^−^ and NH_4_^+^ ions in the groundwater when combined with the sandy soil texture [41]. To satisfy their own growth needs, trees draw water and nutrients from groundwater and deep soil layers [42,43]. Therefore, as the forest ages, *Populus tomentosa*’s ability to acquire deep soil resources through vertical root extension strengthens, significantly reducing its reliance on surface water and nitrogen inputs. Correspondingly, canopy competition effects become more pronounced with increasing stand density, as overlapping crowns mutually shade each other, impeding trees’ capacity to absorb and transport soil moisture and inorganic salts due to diminished transpiration pull [2,44,45,46]. Another possible explanation for the weakening water–nitrogen coupling effect with increasing stand age stems from nutrient limitations caused by prolonged nitrogen input [47]. Specifically, sustained nitrogen addition may trigger secondary effects such as the depletion of soil base cations, enhanced aluminum ion activity, and soil acidification, which have been demonstrated to significantly inhibit forest growth [48,49].

This research also discovered that water–nitrogen coupling enhanced the effect of water inputs on stand growth compared to irrigation alone (Figure 3). Specifically, in the irrigation-only treatment, there were no significant differences in stand *BA*_S_, *V*_S_, and AFP with increasing irrigation amounts (Figure 3a,c,e). However, when comparing the six water–nitrogen treatments, we found that irrigation significantly improved the benefits of *BA*_S_, *V*_S_, and AFP, with particularly noticeable effects at lower fertilization rates (Figure 3b,d,f). According to stand characteristics at the conclusion of the rotation period, a lower fertilization treatment—rather than the maximum fertilization treatment—is the ideal fertilization rate under various irrigation gradients. More development is encouraged in the early stages by high fertilization (Figure 2). Long-term, though, this large fertilizer input results in decreased forest yield in addition to resource waste (Figure 3). Consequently, the forest age effect is the first element we cannot ignore in the intensive cultivation of water and nitrogen, followed by the trees’ irrigation level. We should think about the nutrient variables and the water-to-fertilizer ratio only after the trees’ water needs have been satisfied.

### 3.2. Long-Term Effects of Water–Nitrogen Coupling on Biomass Allocation

The total biomass ratio between the aboveground and belowground portions of the trees stayed comparatively constant throughout the various treatments as they grew and matured (Figure 4a). This supports the Isometric Growth Hypothesis by showing that the aboveground and belowground portions expanded at the same relative growth rate throughout the growth process [50,51,52]. The main limiting factor at the end of the rotation cycle is competition for living space, which progressively overtakes the availability of water and nutrients. According to the Functional Equilibrium Hypothesis and the Optimal Partitioning Hypothesis of biomass allocation, aboveground allocation gradually rises while belowground allocation falls (Figure 6a,c) [25,33,53]. In order to maximize their growth and reproductive benefits, plants typically optimize the distribution of biomass between aboveground and underground parts based on the availability of environmental resources like light, water, and nutrients [7,22,28,32,54,55,56]. Nevertheless, neither high water irrigation nor high fertilizer treatment upset the distribution ratio of the above-ground and below-ground biomass of forest trees, which would finally attain a balanced condition following long-term water and nitrogen coupling measures (Figure 4a). On the one hand, it might be because poplar growth in sandy soils is restricted by water and nitrogen, and the addition of these elements would encourage the development of the underground root system [57,58] (Figure 5b). Furthermore, in order to guarantee steady growth throughout the sapling period, fast-growing tree species need a more robust subterranean structure [39]. However, the accumulation of above-ground biomass is limited since the growing space for the forest trees’ aboveground portion is gradually constricted.

We discovered some minor variations between the various organs, though. For example, plants lower biomass allocation to the higher canopy branches under high water treatment (I20) (Figure 5a). This is mainly because *Populus tomentosa* that receive enough irrigation grow more radially, which results in larger diameters, while their height growth is decreased [59]. Low irrigation treatments, on the other hand, encourage trees to grow vertically in order to compete for additional light resources, which results in more branches developing in the top canopy [60]. In the root system, we did not find that water and nitrogen treatments could regulate the biomass allocation of fine and coarse roots (Figure 5b). Nonetheless, the I20F0 treatment considerably increased the percentage of skeletal root biomass, whereas the high fertilizer treatment (FH) tended to decrease this percentage (Figure 5b). This also suggests that while long-term irrigation can improve the stability of tree root systems, adding nitrogen fertilizer carries the risk of reducing root stability and tree anchoring capacity. Likewise, we saw a definite relationship between the proportion of root systems to branches. The biomass allocation ratio of skeletal roots that serve as subsurface anchors increased in proportion to the aboveground branch allocation ratio (Figure 4b and Figure 5b). Because larger branch systems are more likely to windthrow under windy conditions, the skeletal roots that serve as anchors also grow in an adaptable manner [61]. This further illustrates how functional matching and adaptation in plants are evolutionary processes [62].

### 3.3. Effects of Water–Nitrogen Coupling on WUE and PFP at the End of the Rotation Period

Water scarcity in China has been worse overall in recent years due to climate change, with the North China Plain experiencing an especially severe shortage [63,64]. Poplar plantations should have enough irrigation volume to maintain tree growth without wasting water resources [34,65]. One of the most pressing concerns in the irrigation management of poplar plantations in North China is the rational use of water resources and increasing water usage efficiency. This study’s findings showed that when irrigation volume increases, the stand’s WUE fell (Figure 7 and Figure 8). Notably, the WUE of the stand was not significantly affected by varying nitrogen application gradients (Figure 7 and Figure 8, Table S1). This result contrasts with that of Gu et al. [4], who found that increasing nitrogen treatment can improve winter rapeseed yield and WUE. This discovery may be connected to plant traits and developmental stages. According to earlier research [8,9], *Populus tomentosa* responded significantly to fertilizer and water during the seedling stage, allowing it to quickly acquire growth dominance. But as the forest matures, the trees became less sensitive to nitrogen and water, and the benefits of fertilizer and irrigation on growth and development declined as well (Table 1). Moreover, there was no significant difference in WUE under different fertilization treatments by the end of the rotation period.

PFP, in contrast to WUE, was controlled by fertilization and irrigation, although there was little interaction between them. This suggests that PFP is independently controlled by fertilization and irrigation rather than being impacted by the combined effects of water and nitrogen. At age six, irrigation could encourage PFP improvement, whereas more fertilization caused PFP to decline (Figure 7b). This is consistent with our results of tree growth at 5–6 years of age, indicating that PFP is not always enhanced by the maximal nitrogen supply to encourage wood accumulation. This also suggests that the sandy loam soil at this experimental site has a poor nutrient retention capacity, and that nitrogen fertilizers are prone to leaching, which results in a low partial productivity of nitrogen fertilizers and a lower rate of nitrogen fertilizer utilization by trees. In addition to posing hazards to ecological security, the likelihood of fertilizer leakage is anticipated to increase [66], which means that a high economic investment does not provide a similarly high output.

A reasonable stand density allows trees to make full use of resources including light, water, and nutrients [67]. Excessive stand density causes tree roots to intertwine and compress, resulting in competition for water and nutrients, reducing each tree’s availability for these resources. Furthermore, the mutual shade of tree canopies reduces light availability for understory trees, lowering photosynthesis and decreasing the efficiency of water and nutrient utilization, affecting irrigation efficiency and nitrogen fertilizer partial production. As a result, stand density near the conclusion of the rotation period plays an important role in restricting water and nutrient efficiency, as well as tree growth. For semi-humid sandy short-rotation plantations, discontinuing irrigation and fertilizer at the end of the rotation period may be the most effective management method.

## 4. Materials and Methods

### 4.1. General Description of the Experimental Site

The experimental site is at the State-owned Jiucheng Forest Farm in in Gaotang 

County, Liaocheng City, China (36°48′47″ N, 116°05′25″ E) (Figure S1). *Populus tomentosa* is grown primarily in the North China Plain. The research area has a typical temperate continental semi-arid monsoon environment, with distinct seasons and plenty of sunlight [8]. The total annual sunshine duration is 2651.9 h, with 204 frost-free days. Rainfall is heaviest in July and August, accounting for 544.7 mm on average every year. The average annual temperature is 13.2 °C, with peaks of 41.2 °C and lows of −20.8 °C. The topography of the study site is dominated by plains, and the water table is 6 to 9 m deep [68]. The soil texture is sandy loam with 2.72% clay. Before afforestation, the average soil organic matter content in 0–80 cm was 0.334%, the total nitrogen content was 0.023%, and the available phosphorus content was 4.92 mg·kg^−1^. For further details regarding the physical and chemical properties of the soil, refer to He et al. [8] and Liu et al. [68].

### 4.2. Experimental Design

This study focuses on the triploid Chinese white poplar S86 clone (*Populus tomentosa × Populus bolleana*) × (*Populus alba × Populus glandulosa*). In spring 2016, two-year-old bare-root stem cuttings were chosen for afforestation. The cuttings’ diameter at breast height ranged from 2.12 to 3.51 cm, with an average of 2.68 cm, while their height ranged from 2.60 to 3.62 m, with an average of 3.30 m. The trees were planted equally at a spacing of 2 m × 3 m, yielding a planting density of 1666 trees per hectare. Each tree received 70 g of base fertilizer with N + P_2_O_5_ + K_2_O ≥ 31% (14–12-5) and OM ≥ 15%. In 2016, the drip irrigation system was finished. The drip irrigation belts were placed 30 cm from the tree on both sides, in a two-line per row configuration. In 2017, the experiment with fertilization and irrigation began.

A completely randomized block design was used to establish the experimental layout with nitrogen application rate (F) and irrigation amount (I) as the treatment factors. This resulted in six water–nitrogen treatments (IF) and an additional natural control treatment (CK). The irrigation amount was set based on the quantitative relationship between the growth of *Populus tomentosa* and soil water availability [69]. The trees received irrigation when the soil water potential (SWP) at 20 cm below the dripper, as determined by a tensiometer, reached −20 kPa and −45 kPa (designated as I20 and I45, respectively). The rationale behind these irrigation thresholds was described in He et al. [8]. Based on the growth rhythm and nitrogen need characteristics of *Populus tomentosa*, fertilization rates were set [30]. Three nitrogen application levels were established: 0 (N0), low (NL: 80 kg ha^−1^ in 2017 and 120 kg ha^−1^ from 2018 onwards), and high (NH: 220 kg ha^−1^ in 2017 and 260 kg ha^−1^ from 2018 onwards). The fertilizer choice was urea with a 46% nitrogen content and N fertilizer—the urea nitrate solution was injected directly into a water-driven injector (MixRite Model 2504, Tefen, Israel). According to the biological characteristics of *Populus tomentosa*, which grows rapidly at first and then slows down, the first three applications accounted for 3/5 of the annual fertilization amount, while the last three applications accounted for 2/5 of the annual fertilization amount, with each application interval lasting approximately 20 days. Additionally, a control treatment (CK) without irrigation and fertilization was established. To avoid the influence of light and soil heterogeneity, each block was arranged longitudinally from north to south, with seven treatment plots per block, resulting in a total of five replicates and 35 plots. Each plot consisted of eight rows of trees, with four trees per row, and the middle four rows were designated as experimental trees, while the two outermost rows on the east and west sides served as protective rows. Furthermore, 50 cm deep plastic sheets were buried between adjacent plots within the same block to prevent the movement of water and nitrogen. Chemical agents were used regularly for weed and pest control. The amounts of irrigation and fertilization applied between 2017 and 2021 are shown in Table 2.

### 4.3. Growth Measurement and Biomass Acquisition

#### 4.3.1. Acquisition of Forest Growth Data

From 2016 to 2021, during the growth cessation period after leaf fall (October), the diameter at breast height (DBH) of the sample trees in each experimental plot within the forest was measured using a diameter tape. These data were then used to determine the stand basal area (*BA*_S_, m^2^ ha^−1^) at the end of each year. The height of the sample trees in each plot was measured using a laser hypsometer (Vertex IV, Haglöf, Länsele, Sweden). The annual increment in diameter at breast height (ΔDBH) and the annual increment in tree height (Δ*H*) were calculated using the following formulas:

(1) ΔDBH=DBH2−DBH1

(2) ΔH=H2−H1

Individual tree volume is calculated using the binary volume database for *Populus tomentosa* compiled by Chen [70]:

(3) $$Va=0.5134\times H^{0.827}\times {\left(DBH/100\right)}^{1.9954}$$

DBH1 and DBH2 and *H*1 and *H*2 reflect the measured values of diameter at breast height and tree height over two years, respectively. *V*a represents the individual tree volume, *H* is the tree height, and DBH is the diameter at breast height.

The individual tree volume (*V*a) multiplied by the number of trees per hectare yields the stand volume per hectare (*V*_S_). Then, dividing the stand volume per hectare by the number of planting years gives the annual forest productivity (AFP).

#### 4.3.2. Acquisition and Calculation of Forest Biomass Data

In early March 2022, before the trees had sprouted and matured, three sample trees (with breast height diameters close to the plot average) were chosen from the I20FH, I20F0, I45FH, and CK plots for destructive biomass sampling. After the sample trees were felled, their total height and height to the first branch were measured, and they were classified into three strata based on crown length: upper, middle, and lower. The sample trees’ root systems were manually excavated in a 2 m × 3 m area, centered on the stump, to a depth of 1 m, yielding 6 m^3^ of excavated soil. Samples from each tree were classified as upper canopy branches, lower canopy branches, trunks, root stumps, skeleton roots (>5 mm), coarse roots (2–5 mm), and fine roots (<2 mm). Samples of each part were brought back to the laboratory and dried at 65 °C until the mass was unchanged. The moisture content of each part was calculated from the samples and converted to the dry weight of each organ. Aboveground biomass (AGB) is the combined biomass of branches and stems, whereas belowground biomass (BGB) is the combined biomass of rootstocks, skeletal roots, coarse roots, and fine roots. The total biomass of a plant (TB) is the sum of its constituent elements. The allocation ratio for each organ is computed using the biomass of the various organs, belowground biomass, and total plant biomass.

(4) TMR=SB/TB

(5) BMR=BB/TB

*SB*, *BB*, and *TB* represent the dry weight of biomass (kg) for the trunk, branches, and total plant, respectively.

In October 2017, four sample trees (DBH values comparable to the plot average) were chosen from the I20H and CK plots for destructive biomass sampling. In October 2018, three sample trees were chosen from the I20FH, I20F0, I45FH, and CK plots for destructive biomass sampling. A total of 32 sample trees were collected in 2017, 2018, and 2022. The models y = ax + b and y = ax^2^ + bx + c were developed based on data on total plant biomass, aboveground and belowground biomass, and tree breast height diameter. Figure 7 and Figure S2 illustrate the findings of these models.

#### 4.3.3. Irrigation Water Use Efficiency and Nitrogen Fertilizer Partial Productivity

The irrigation water use efficiency (WUE, m^3^·ha^−1^·mm^−1^) was determined using Equation (6). This study calculated the total irrigation water use efficiency since the initiation of irrigation in 2017. In this context, *V*S_total_ (m^3^·ha^−1^) represents the forest stand volume of the I20F0, I45F0, and CK treatments at the end of 2021, and *IA* (mm) is the total irrigation amount applied during the research period from 2017 to 2021.

(6) $$WUE={VS}_{total}/IA$$

We used R^2^ as the metric for model goodness-of-fit, where a higher R^2^ indicates better agreement between predicted and measured values [71,72]. The univariate quadratic regression and linear regression models for biomass–DBH yielded *R*^2^ values of 0.9274 and 0.8950, respectively (Figure S2). Given the average DBH per treatment plot at the end of 2021, we selected the univariate quadratic regression model to calculate total biomass per plant under water–nitrogen coupling treatments. Equation (7) was used to evaluate the partial factor productivity of nitrogen fertilizer (PFP, kg·kg^−1^). This study calculated the PFP since its application in 2017. *TB*_total_ (kg·ha^−1^) is the total biomass per hectare determined by the model for I20FL, I20FH, I45FL, and I45FH, and *T* (kg·ha^−^) is the total quantity of nitrogen fertilizer applied over the research period from 2017 to 2021.

(7) $$PFP={\;TB}_{total}/T$$

### 4.4. Statistics and Analysis of Data

The data were first organized using Excel 2019. A Levene’s test was applied to verify the homogeneity of variances, and a Kolmogorov–Smirnov test was used to check for normality. The data were converted using the natural logarithm (ln) since the biomass allocation ratios, TMR, and AGB\RGB of the upper and intermediate canopy branches did not satisfy the requirements for an ANOVA. Data on biomass allocation and tree growth were subjected to a one-way ANOVA to examine the differences between water and nitrogen treatments and the control treatment. The effects of irrigation (I), nitrogen application (F), and their interaction (I × F) under water–nitrogen coupling (IF) treatment were examined using a two-way ANOVA on tree growth data, WUE, and PFP. Based on significant differences from CK, multiple comparisons were performed using a Duncan’s test (α = 0.05).

Using IBM SPSS Statistics software (SPSS25, IBM Inc., Chicago, IL, USA), a Spearman’s correlation analysis and path analysis were performed using the irrigation level (W) and fertilization level (N) from treatments I20F0, I20FL, I20FH, I45F0, I45FL, and I45FH in 2021 as independent variables (*X*_j_) and growth indicators, stock volume, forest productivity, forest biomass, water use efficiency, and nitrogen fertilizer partial productivity as dependent variables (*Y*_j_). Standardized regression coefficients were interpreted as direct path coefficients (ci), since this study focuses only on the direct effects of X_j_ on Y_j_. Origin software (Origin 2023, Origin Lab Corporation, Northampton, MA, USA) was used to plot the graphs.

## 5. Conclusions

This study showed that there is a considerable age effect in the effects of nitrogen and water treatments on short-rotation *Populus tomentosa* forestry development. The growth of DBH at the age of two years was greatly aided by the coupling effect of water and nitrogen at the individual level; the I20FH treatment dramatically increased DBH by 37.30%. However, beyond the age of three, this encouraging influence rapidly faded. *Populus tomentosa*’s aboveground–belowground biomass allocation ratio remains unchanged when water–nitrogen coupling is applied. The aboveground biomass allocation ranged from 5.16 to 6.62 times that of the belowground biomass. As the stand grows, the aboveground allocation increases and the belowground allocation diminishes toward the conclusion of the rotation period. Notably, the allocation ratio of branches to skeletal roots changes in a coordinated manner, indicating the evolutionary approach of plant functional adaptation. The irrigation level had a negative impact on the WUE of 6-year-old *Populus tomentosa*, with the I20 treatment reducing it by 88.79% compared to the I45 treatment. Additionally, due to the poor nutrient retention capacity of the sandy loam soil at the experimental site, nitrogen fertilizer is prone to leaching. As the fertilization rate increased, the PFP decreased by 130.95% to 132.86%.

Thus, it is recommended to implement full irrigation (irrigate when soil water potential reaches −20 kpa) and nitrogen fertilization for high-density, short-rotation *Populus tomentosa* plantations during the sapling stage (ages 1–3) in semi-humid sandy loam regions, while ceasing fertilization and irrigation management during the 4–6 year growth period. This strategy not only reduces resource waste, but it also maintains stand productivity. This work provides both a theoretical foundation and practical assistance for accurate water and fertilizer management in short-rotation plantations.

## Figures and Tables

**Table 1:** The one-way ANOVA results of the differences in growth indicators between the water and nitrogen treatment (IF) and the control treatment (CK). The two-way ANOVA assessed the effects of irrigation level, fertilization level, and the interaction between irrigation and fertilization on various growth indicators. In the table, ΔDBH represents the annual increment of individual tree diameter at breast height, Δ*H* denotes the annual increment of individual tree height, *BA*_S_ stands for the stand basal area in different years, *Vs* indicates the stand volume in different years, and AFP refers to the average forest productivity in different years. Bold values indicate statistically significant effects.

Indices	Source of Variation	Stand Ages (Years Old)									
		2		3		4		5		6	
		*F*-Value	*p*-Value	*F*-Value	*p*-Value	*F*-Value	*p*-Value	*F*-Value	*p*-Value	*F*-Value	*p*-Value
ΔDBH (cm)	IF vs. CK	3.886	**0.006**	1.654	0.17	0.459	0.832	0.759	0.608	1.133	0.369
	I	15.88	**0.001**	0.387	0.061	0.009	0.926	0.063	0.804	0.106	0.748
	F	0.494	0.616	1.378	0.271	0.653	0.53	0.527	0.597	0.501	0.612
	I × F	0.463	0.635	0.147	0.864	0.021	0.979	1.278	0.297	1.848	0.179
Δ*H* (m)	IF vs. CK	1.247	0.313	1.177	0.347	0.727	0.632	1.230	0.321	0.865	0.533
	I	0.694	0.413	0.138	0.714	1.468	0.238	0.021	0.886	3.035	0.094
	F	0.746	0.485	2.32	0.12	0.898	0.421	3.711	**0.039**	0.343	0.713
	I × F	0.183	0.834	0.107	0.899	0.387	0.683	0.176	0.840	0.069	0.934
*BA_S_* (m^2^·ha^−1^)	IF vs. CK	2.631	**0.038**	2.893	**0.025**	2.191	0.074	2.197	0.073	1.996	0.100
	I	8.628	**0.007**	9.877	**0.004**	6.445	**0.018**	9.227	**0.006**	8.814	**0.007**
	F	0.714	0.500	0.355	0.705	0.426	0.658	0.281	0.757	0.632	0.540
	I × F	0.633	0.539	0.678	0.517	0.449	0.644	1.548	0.233	0.178	0.838
*V_S_* (m^3^·ha^−1^)	IF vs. CK	2.302	0.062	2.840	**0.027**	2.272	0.065	2.325	0.060	1.841	0.127
	I	6.229	**0.020**	9.662	**0.005**	6.878	**0.015**	9.207	**0.006**	7.894	**0.010**
	F	0.893	0.423	0.288	0.753	0.405	0.617	0.287	0.753	0.288	0.752
	I × F	0.472	0.629	0.608	0.553	0.516	0.603	1.522	0.239	0.241	0.788
AFP (m^3^·ha^−1^·yr^−1^)	IF vs. CK	2.302	0.062	2.840	**0.027**	2.272	0.065	2.325	0.060	1.841	0.127
	I	6.229	**0.020**	9.662	**0.005**	6.878	**0.015**	9.207	**0.006**	7.894	**0.010**
	F	0.893	0.423	0.288	0.753	0.405	0.617	0.287	0.753	0.288	0.752
	I × F	0.472	0.629	0.608	0.553	0.516	0.603	1.522	0.239	0.241	0.799

**Table 2:** Irrigation amount and fertilization amount under different treatments from 2017 to 2021.

Treatment	Irrigation When SWP at 20 cm Depth (kPa)	Irrigation Amount (mm·ha^−1^·year^−1^)					Fertigation Amount (kg ha^−1^ year^−1^N)				
		2017	2018	2019	2020	2021	2017	2018	2019	2020	2021
I20F0	−20	347.40	455.73	291.67	309.90	364.57	0	0	0	0	0
I20FL	−20	347.40	455.73	291.67	309.90	364.57	80	120	120	120	120
I20FH	−20	347.40	455.73	291.67	309.90	364.57	220	260	260	260	260
I45F0	−45	16.67	0.00	25.52	51.10	76.78	0	0	0	0	0
I45FL	−45	16.67	0.00	25.52	51.10	76.78	80	120	120	120	120
I45FH	−45	16.67	0.00	25.52	51.10	76.78	220	260	260	260	260
Control	Non-irrigation	0.00	0.00	0.00	0.00	0.00	0	0	0	0	0

Note: I20 and I45 indicate irrigation when the soil water potential reaches −20 kPa and −45 kPa, respectively, while F0, FL, and FH represent three levels of fertilization: no fertilization, low fertilization rate, and high fertilization. The irrigation volume represents the total amount of water applied under the irrigation scenario of the current year. Since the irrigation volume for fertigation is relatively small, it was not calculated in this study.

## Data Availability

The original contributions presented in this study are included in the article/Supplementary Materials. Further inquiries can be directed to the corresponding author(s).

## Supplementary Materials

The following supporting information can be downloaded at: https://www.mdpi.com/article/10.3390/plants14121833/s1, Table S1. Significance of correlations between irrigation and fertilization gradients and forest growth indicators and forestland indicators. In the table, W represents irrigation level, N represents fertilization level, *BA*_S_ represents tree basal area, H represents mean tree height, *V*_S_ represents stand volume in different years, TGB represents forestland biomass, AFP represents average forestland productivity, WUE represents water use efficiency of irrigation, PFP represents partial factor productivity of nitrogen fertilizer. *** indicates *p* < 0.001; ** indicates *p* < 0.01; * indicates *p* < 0.05. Figure S1. The positions of the experimental site and experimental plantations [colored areas in (a,b)]. The boundaries of our experimental plantation are indicated by red color (c). Figure S2. Univariate quadratic regression (a) and linear regression model (b) for total tree biomass and diameter at breast height. The pink shading represents the 95% prediction band, and the gray shading represents the 95% confidence band (n = 32).
