# Supplementary material for: Growth and Biomass Distribution Responses of Populus tomentosa to Long-Term Water–Nitrogen Coupling in the North China Plain
Source: Plants (Basel). 2025 Jun 14;14(12):1833. doi: 10.3390/plants14121833 (PMC12196843; doi:10.3390/plants14121833)
Supplement: Supplementary file 1 [file plants-14-01833-s001.zip › plants-3673566-supplementary.pdf]

Growth and Biomass Distribution Responses of *Populus tomentosa* to Long-Term Water–Nitrogen Coupling in the North China Plain

Yafei Wang [1,†], Juntao Liu [1,2,†], Yuelin He [3], Wei Zhu [4], Liming Jia [1,*] and Benye Xi [1,*]

[1] Key Laboratory of Silviculture and Conservation of the Ministry of Education, College of Forestry, Beijing Forestry University, Beijing 100083, China

[2] Guangdong Provincial Key Laboratory of Silviculture, Protection and Utilization/Guangdong Academy of Forestry, Guangzhou 510520, China

[3] National Key Laboratory for Development and Utilization of Forest Food Resources, Zhejiang A&F University, Hangzhou 311300, China

[4] College of Biological and Environmental Engineering, Jingdezhen University, Jingdezhen 333400, China

Supplementary materials

Table S1. Significance of correlations between irrigation and fertilization gradients and forest growth indicators and forestland indicators. In the table, W represents irrigation level, N represents fertilization level, BA_S represents tree basal area, H represents mean tree height, V_S represents stand volume in different years, TGB represents forestland biomass, AFP represents average forestland productivity, WUE represents water use efficiency of irrigation, PFP represents partial factor productivity of nitrogen fertilizer. *** indicates P<0.001; ** indicates P<0.01; * indicates P<0.05.

Parameters	W	N	*BAs*	*H*	*Vs*	TGB	AFP	WUE	PFP
W	***								
N		***							
BAs	**		***						
H				***					
Vs	**		***		***				
TGB	**		***		***	***			
AFP	**		***		***	***	***		
WUE	***							***	
PFP		**							***

* Corresponding author, E-mail:jlm@bjfu.edu.cn

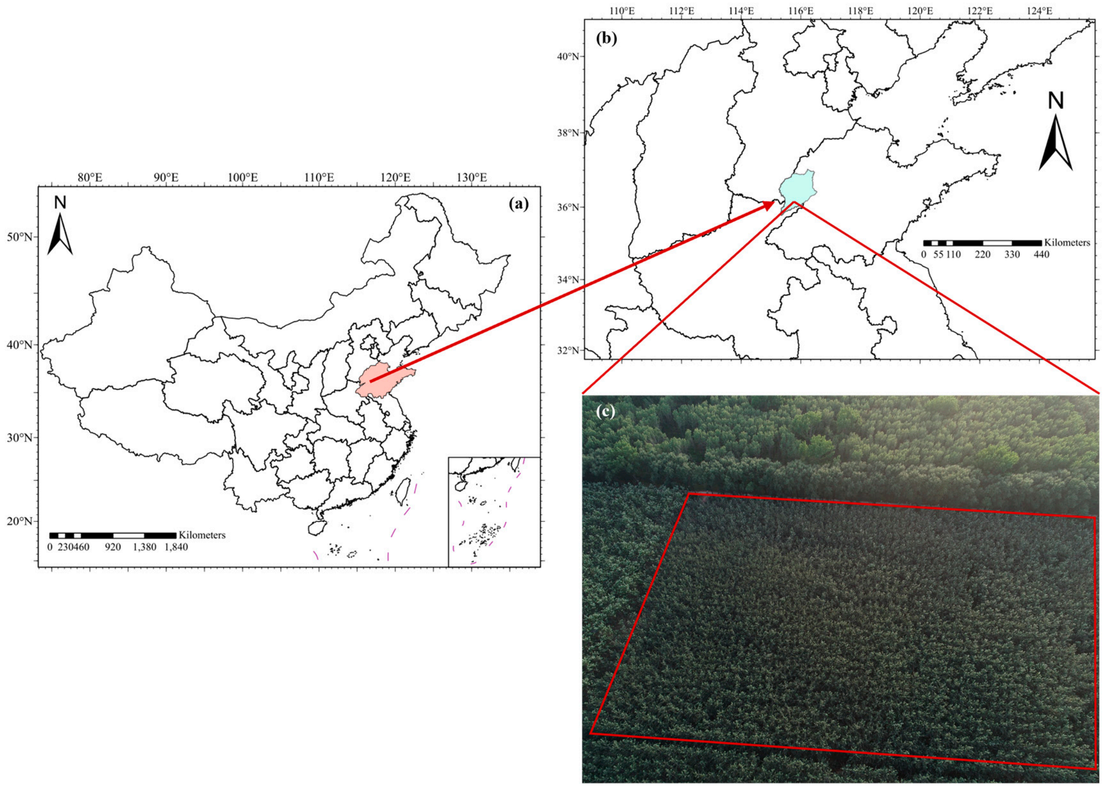

Figure S1. The positions of the experimental site and experimental plantations [colored areas in(a,b)]. The boundaries of our experimental plantation are indicated by red color (c).

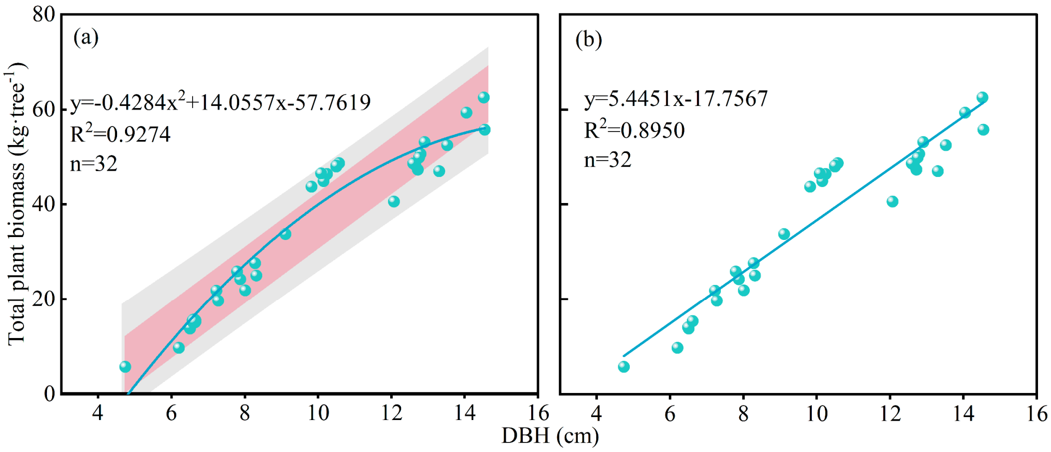

Figure S2. Univariate quadratic regression (a) and linear regression model (b) for total tree biomass and diameter at breast height. The pink shading represents the 95% prediction band, and the gray shading represents the 95% confidence band (n=32).

* Corresponding author, E-mail:jlm@bjfu.edu.cn
